# Overweight, resting heart rate, and prediabetes/diabetes: A population-based prospective cohort study among Inner Mongolians in China

**DOI:** 10.1038/srep23939

**Published:** 2016-03-31

**Authors:** Shao Yan Zhang, Jia Hui Wu, Jing Wen Zhou, Zhu Liang, Qiao Yan Qiu, Tian Xu, Ming Zhi Zhang, Chong Ke Zhong, Wei Jiang, Yong Hong Zhang

**Affiliations:** 1 Department of Epidemiology, School of Public Health, Medical College of Soochow University, Suzhou 215123, Jiangsu, China; 2 Jiangsu Key Laboratory of Preventive and Translational Medicine for Geriatric Diseases, School of Public Health, Soochow University, Suzhou 215123, People’s Republic of China

## Abstract

We aimed to investigate the cumulative effect of overweight and resting heart rate on prediabetes/diabetes incidence in an 10-year follow-up study in Inner Mongolians. Among 1729 participants who were free from prediabetes and diabetes at baseline, 503 and 155 subjects developed prediabetes and diabetes, respectively. We categorized the participants into 4 subgroups according to overweight and resting heart rate status. The multivariate-adjusted OR (95% CI) in normal weight with heart rate ≥80 bpm, overweight with heart rate <80 bpm, and overweight with heart rate ≥80 bpm were 1.24 (0.95–1.61), 1.83 (1.29–2.61), 2.20 (1.41–3.45) for prediabetes and 1.52 (0.97–2.40), 3.64 (2.21–6.01), 4.61 (2.47–8.61) for diabetes, respectively, compared with normal weight with heart rate <80 bpm. The area under ROC curve (AUC) for the prediction of diabetes incidence for a model containing overweight and resting heart rate, along with conventional factors (AUC = 0.751), was significantly (P = 0.003) larger than the one containing only conventional factors (AUC = 0.707). Our study indicated that overweight was an independent risk factor of prediabetes and diabetes, and overweight with faster resting heart rate might further increase the risk of prediabetes and diabetes.

Cardiovascular disease has become the leading causes of serious, long-term disability and death worldwide^1^. Diabetes is recognized as a major risk factor for cardiovascular disease. The prevalence of diabetes is high and is increasing rapidly in China^2^,^3^, a development that has followed the change in lifestyle and dietary habits. Prediabetes refers to a condition progressing to diabetes and includes the two abnormalities: impaired fasting glucose(IFG) and impaired glucose tolerance(IGT)^4^. A recent national survey of Chinese adults showed that the prevalence of diabetes and prediabetes were 9.7% and 15.5%, and China may bear a higher diabetes-related burden than any other country^3^,^5^.

Overweight and obesity is the most common and a strong modifiable risk factor for diabetes^6^,^7^,^8^. The prevalence of overweight and obesity has increased throughout the world, including Asia^9^. The China National Nutrition and Health Survey (CNNHS)^10^ showed that the prevalence of overweight and obesity in Chinese adults amounts to 22.8% and 7.1%, respectively. It is also suggested that heart rate may play an important role in diabetes development^11^,^12^. However, no study has specifically evaluated the cumulative effect of overweight and resting heart rate on risk of diabetes in an Inner Mongolian population (an ethnic minority in China). In the present study, we analyzed the association among overweight, resting heart rate, and prediabetes/diabetes in an over 10-year follow-up study among Inner Mongolians from China.

## Methods

### Study participants

The study was established to evaluate potential risk factors for chronic diseases during the period July 2002 to September 2003. The methods for study participants recruitment and baseline data collection have been described elsewhere^13^. Briefly, study participants aged 20 years or more were recruited from 32 villages in 2 adjacent townships located in the counties of Kezuohou Banner and Naiman Banner in Inner Mongolia. The majority of the residents in the investigation field are Mongolians who have lived there for many generations and maintain a traditional diet and lifestyle. A total of 3475 Mongolian people ≥20 years of age live in these villages. Among them, 889 people were excluded because they refused to participate or had cardiovascular diseases or endocrine diseases or anxiety neurosis. Finally, a total of 2589 people (Men: 1064, Women: 1525) aged 20 to 84 years old participated in the study at baseline. This study was approved by the Soochow University Ethics Committee in China. Written informed consent was obtained for all study participants. The methods were carried out in accordance with relevant guidelines and regulations.

This cohort was re-investigated in 2013. The present study consisted of 2250 participants free of diabetes and IFG at baseline, who successfully completed a baseline fasting plasma glucose (FPG) test, and cases who registered the baseline address. Of those individuals, 1729 participated in the follow-up, including fasting plasma glucose (FPG) test and the oral glucose tolerance test (OGTT), 249 were dead and 272 were lost to follow up. Those who attended the follow-up examination were more likely to be female, younger, lower blood pressure level, lower rate of drinking (P < 0.05) than those who did not attend, but there were no significant differences in BMI, resting heart rate, smoking, baseline FPG, homeostasis model assessment-insulin resistance (HOMA-IR) and hyperlipidemia (P > 0.09).

### Data collection

Trained staff interviewed participants in Chinese using a standard questionnaire to obtain information on demographic characteristics, medical history, and lifestyle risk factors. Cigarette smoker was defined as having smoked at least one cigarette per day for 1 year or more. Alcohol drinking was defined as having consumed at least 50 g alcohol per day for 1 year or more. Three blood pressure measurements were taken for each participant while participants were seated using a mercury sphygmomanometer according to a standard protocol^14^. The first and fifth Korotkoff sounds were recorded as systolic and diastolic blood pressure, respectively. The mean of these three blood pressure measurements was used for data analysis. Resting heart rates were measured three times by stethoscope at the apex of the heart and counted for a 60-second interval; 1 min apart, following at least 30-min rest. Body weight and height were measured by trained staff using a balance beam scale after subjects removed their shoes and were wearing light clothing. Body mass index (BMI) was calculated as weight in kilograms divided by the square of the height in meters (kg/m^2^). Overweight was defined as BMI ≥ 25 kg/m^2^.

Overnight fasting blood samples were obtained to measure cholesterol, blood glucose and insulin. Plasma and serum samples were frozen at −80 °C until laboratory testing. A modified hexokinase enzymatic method was applied to test plasma glucose levels^15^. Serum insulin was measured using a radioimmunoassay method, and the HOMA-IR score was calculated [FPG(mmol/l) × fasting serum insulin (mU/l)/22.5]. Total cholesterol(TC), high-density lipoprotein cholesterol(HDL-C), and triglycerides(TG) were assessed enzymatically using a Beckman Synchron CX5 Delta Clinical System (Beckman Coulter, Inc; Fullerton, CA) with commercial reagents^16^. Low-density lipoprotein cholesterol levels were calculated using the Friedewald equation for participants who had <400 mg/dl triglycerides^17^. Hyperlipidemia was defined as total cholesterol ≥5.72 mmol/L and/or triglycerides ≥1.70 mmol/L and/or LDL-C ≥3.64 mmol/L and/or high-density lipoprotein cholesterol <0.91 mmol/L.

### Follow up and ascertainment of outcomes

Trained staff interviewed either the participants or their relatives, if participants were dead or unable to communicate, and completed a medical status questionnaire. If a participant or their relatives reported that diabetes occurred during the period baseline survey to follow up, the staff contacted the subject’ general practitioner and reviewed hospital records or death certificate to confirm. All other participants alive were instructed to maintain their usual physical activity and diet for at least 3 days before the OGTT according to a standard protocol. After at least 10 hours of overnight fasting, fasting venous blood glucose was sampled and standard 75 g glucose solution were given. Blood samples were drawn at 120 minutes after the glucose load to measure glucose concentrations. Plasma glucose level was also measured with the use of the hexokinase enzymatic method. Incident diabetes was defined based on 1999 World Health Organization (WHO)^18^ criteria (≥7.0 mmol/l fasting or ≥11.1 mmol/l 2-h glucose) or validated physician diagnosis or the use of antidiabetic medication at any investigation or diagnosed as diabetes in the medical records or death certificate. Incident prediabetes was also defined according to the 1999 WHO diagnostic criteria (1) IFG (≥6.1 mmol and <7.0 mmol/l fasting and <7.8 mmol/l 2-h glucose), (2) IGT (<7.0 mmol/l fasting and ≥7.8 mmol/l and <l 11.1 mmol/l 2-h glucose).

### Statistical analysis

Plasma glucose was categorized into three groups: Normal glucose tolerance, prediabetes, diabetes. Resting heart rate was grouped comparing the upper tertile (≥80 bpm) to the bottom 2 tertiles (<80 bpm). We categorized all participants into four subgroups: normal weight with heart rate <80 bpm, overweight with heart rate <80 bpm, normal weight with heart rate ≥80 bpm, and overweight with heart rate ≥80 bpm. Conventional cardiovascular risk factors among four subgroups were compared using analysis of variance for continuous variables and chi-squared tests for categorical variables. We use multivariate logistic regression model to compute odds ratios (ORs) of prediabetes and diabetes among four subgroups by adjusting the important confounding factors including age, sex, smoking status, drinking status, systolic blood pressure, diastolic blood pressure, HOMA-IR and hyperlipidemia. We set a multiplicative interaction term of overweight and resting heart rate in multivariate logistic regression model and tested its effect on prediabetes and diabetes incidence, independent of overweight, resting heart rate, and other confounding factors. We also assessed the discriminatory value of overweight/heart rate by computing the area under receiver operating characteristic curves (AUC) and comparing a model including only conventional risk factors with a model including overweight and resting heart rate subgroup, in addition to conventional risk factors. All P values were 2-tailed, and a significance level of 0.05 was used. All statistical analyses were conducted using SAS statistical software (version 9.2) and R statistical software (version 2.15).

## Results

Among 1729 people, 503 progressed to prediabetes and 155 were classified as having type 2 diabetes. Of 155 participants with diabetes during the follow-up, 25 were diagnosed before the follow-up examination, and the remaining 130 were classified as having diabetes on the basis of the FPG and OGTT. The cumulative incidence rates of prediabetes and diabetes were 29.1% and 9.0%, and the incidence density was 2711 and 835 per 100 000 person-years, respectively.

The baseline characteristics of participants by the four study subgroups are shown in Table 1. Conventional diabetes risk factors, such as age, sex, smoking, drinking, blood pressure, lipids and HOMA-IR, were significantly different among the four subgroups. Overweight participants in either heart rate group had lower rate of smoking and had higher levels of systolic blood pressure, diastolic blood pressure, TC, TG, LDL-C, HOMA-IR and lower HDL-C. Normal weight and overweight participants with heart rate ≥80 bpm tended to be women and had lower rate of drinking.

During 10 years of follow-up, the cumulative incidence rates among four subgroups were 25.6, 29.9, 36.2, 40.7% for prediabetes, and 6.0, 8.2,17.6, 19.5% for diabetes, respectively (P < 0.001), and the overweight subjects with heart rate ≥80 bpm had highest cumulative incidence rate of prediabetes and diabetes.

Table 2 presents the age-sex adjusted and multivariate-adjusted ORs and 95% confidence intervals (95% CIs) of prediabetes and diabetes according to the overweight and resting heart rate status. Compared with normal weight subjects with heart rate <80 bpm, the age-sex adjusted ORs (95% CIs) in normal weight/heart rate ≥80 bpm, overweight/heart rate <80 bpm and overweight/heart rate ≥80 bpm subgroups were 1.34 (1.04–1.74), 2.12 (1.51–2.97), 2.64 (1.72–4.06) for prediabetes and 1.77 (1.14–2.77), 4.91 (3.05–7.92), 6.61 (3.70–11.83) for diabetes, respectively. After adjusting for other confounding factors, the ORs (95% CIs) in normal weight/heart rate ≥80 bpm, overweight/heart rate <80 bpm and overweight/heart rate ≥80 bpm subgroups were 1.24 (0.95–1.61), 1.83 (1.29–2.61), 2.20 (1.41–3.45) for prediabetes and 1.52 (0.97–2.40), 3.64 (2.21–6.01), 4.61 (2.47–8.61) for diabetes, respectively, compared with the reference group. Overweight subjects with heart rate ≥80 bpm were at a highest risk of prediabetes and diabetes. We also analyzed the independent effects of overweight and resting heart rate on prediabetes and diabetes incidence risk. The multivariable-adjusted ORs (95% CIs) of prediabetes/diabetes for overweight and resting heart rate were 1.82 (1.37–2.42)/3.39 (2.27–5.08) and 1.23 (0.97–1.56)/1.43 (0.98–2.08), respectively. A sensitivity analysis including subjects who lost of follow up and were classified as being normal glucose tolerance cases yielded similar findings for the above associations (data not shown). No significant interaction was detected between overweight and resting heart rate on the prediabetes or diabetes risk (all P > 0.05).

**Figure 1:**
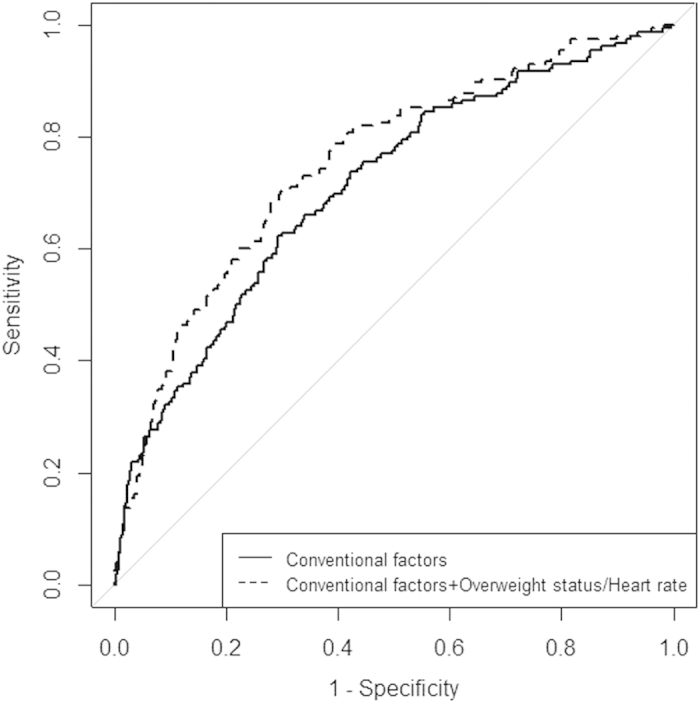
Area under the curve for the prediction of diabetes incidence for baseline conventional risk factors and for the addition of overweight status/resting heart rate. Risk factors in the conventional model include age, sex, smoking, drinking, blood pressure, hyperlipidemia and HOMA-IR.

The AUC for the model including only the conventional risk factors achieved reasonable discrimination with AUC of 0.605 for prediabetes and 0.707 for diabetes, respectively. After adding overweight and resting heart rate status subgroup, the discriminatory value marginally improved by 0.016 (AUC = 0.621, P = 0.095) for prediabetes and 0.044 (AUC = 0.751, P = 0.003) for diabetes (Fig. 1).

## Discussion

In this population-based prospective cohort study among Inner Mongolian population, overweight subjects with heart rate <80 bpm and overweight subjects with heart rate ≥80 bpm were at a significantly higher risk for prediabetes and diabetes than normal weight subjects with heart rate <80 bpm. Overweight subjects with faster resting heart rate were at a highest risk in this population. Overweight was an independent risk factor for prediabetes and diabetes in this study, while resting heart rate was not independently associated with prediabetes and diabetes. Our study is the first one to examine the cumulative effect of overweight and resting heart rate on prediabetes and diabetes incidence in Inner Mongolians, an ethnic minority in China. Compared with previous studies analyzing overweight and resting heart rate separately with diabetes risk, the novelty of this study is to combine these two factors as a new variable and test the cumulative effect of two ones.

It has been recognized that obesity is an important risk factor of diabetes incidence, independent of hypertension, hyperlipidemia, and other conventional risk factors. The Finnish Twin Cohort Study of 23,585 adult Finnish twins showed that overweight and obesity were independently associated with an increased risk of diabetes compared with normal weight participants^19^. The HRs (95% CIs) were 2.96 (2.59–3.39), 6.80 (5.62–8.22) and 13.64 (9.28–20.05) for overweight, obese and morbidly obese participants, respectively. One meta-analysis by Hartemink *et al*. reported that diabetes risk increased by 1.18 (95% CI: 1.16, 1.20) per unit of BMI and assumes a linear relation between BMI and the relative risk of diabetes^20^, another meta-analysis of prospective cohort study also found the RR of diabetes for obese persons compared to those with normal weight was 7.28, 95% CI: 6.47, 8.28 and for overweight was 2.92, 95% CI: 2.57, 3.32 8,21. In China, some prospective cohort studies indicated that overweight and obesity defined by BMI were independent predictors of diabetes in the Han population^7^,^21^. There is evidence showing that obesity adversely affects various underlying causes of diabetes, including increased production of nonesterfied fatty acids, adipokines/cytokines, reduced levels of adiponectin and mitochondrial dysfunction that compromise β-cell function^22^.

A very limited number of prospective studies have reported conflicting data on the relationship between heart rate and diabetes risk^11^,^12^,^23^,^24^,^25^. In the Australian Diabetes Obesity and Lifestyle prospective study^12^, participants with a heart rate >/=80 b min(−1) had a 89% higher risk of developing diabetes than those with a heart rate <60 b min(−1). This risk was highly significant, particularly in non-obese men (461%), but not in their obese counterparts or in women. An over 25-year follow-up of the Chicago Heart Association Detection Project in Industry^26^ showed that higher baseline resting heart rate is associated with diabetes claims and mortality in older age and is only due in part to BMI and concurrently measured glucose. Heart rate is predominantly determined by the sympathetic outflow to the heart and modulated by vagal inputs, and sympathetic activation has been demonstrated to contribute to insulin resistance^27^. Therefore, it is reasonable to speculate that sympathetic activation is the mechanistic link between elevated heart rate and increased diabetes risk. Although high resting heart rate alone is not an independent risk factor of prediabetes and diabetes incidence in our study, the cumulative incidence of prediabetes and diabetes for those with heart rate ≥80 bpm/overweight was 40.7% and 19.5%, and appeared higher than the other categories. Logistic regression model analysis indicated that overweight subjects with heart rate ≥80 bpm had the highest risk of prediabetes and diabetes, with a 2.20-fold and 4.61-fold increased risk compared with normal weight and heart rate <80 bpm, respectively. High resting heart rate seems to amplify the effect of overweight on prediabetes, and the effect on diabetes seems to be further amplified. These findings implied that coexistence of overweight and high resting heart rate increased the risk of hyperglycemia, especially diabetes. Thus, individuals with relatively high resting heart rate should maintain normal weight to reduce diabetes risk in our study population.

Our findings have an important preventive meaning for diabetes among population. At present, diabetes gradually causes micro- and macro-vascular complications and results in an enormous economic burden to society^28^. In addition, prediabetes has been identified as risk factors for overt diabetes and cardiovascular disease^29^. Therefore, it is obvious that the early prevention and intervention is the most effective strategy. The public health measures, such as healthful diet, exercising regularly, controlling weight, and clinic-based diabetes screening for the early detection of hyperglycemia may be effective in lowering diabetes risk and diabetes-related complications in the general population.

This study has several strengths that deserve mention. To our knowledge, it is the first prospective study to examine the association among overweight, resting heart rate, and (pre)diabetes in the Mongolian population, a minority in China. The study participants were homogeneous, because they shared a same genetic background and had similar environmental exposures. The data were collected with rigid quality control and important covariables were measured and controlled in the analysis. The oral glucose tolerance tests were performed in the study population during the follow up period. Therefore, the incidence of (pre)diabetes could not be underestimated. In addition, our follow-up time was relatively long, which enabled us to minimise bias from confounding factors. However, there are also some limitations in the present study. First, we only examined fasting plasma glucose and did not conduct oral glucose tolerance test at baseline. The studies^30^ have showed that some individuals with normal FPG may have impaired glucose tolerance based on oral glucose tolerance tests data. Therefore, the subjects with impaired glucose tolerance at baseline were not excluded during the follow-up. It is also important to note possible bias in our results given that baseline characteristics were different between attendees and non-attendees. However, we did adjust for these variables in our multivariate analyses and the sensitivity analysis yielded similar findings. Second, considering our sample size is not very large, we did not stratify participants into those with obesity and those with overweight. Therefore, this finding did not differentiate the associations of obesity/heart rate ≥80 bpm and overweight/heart rate ≥80 bpm with prediabetes or diabetes. Finally, information on BMI and heart rate status were recorded only once at baseline, therefore, intraindividual variation during the follow-up could not be assessed.

In summary, we found that overweight was an independent risk factor for prediabetes and diabetes, and overweight with faster resting heart rate might further increase the risk of prediabetes or diabetes among Inner Mongolians.

## Additional Information

**How to cite this article**: Zhang, S. Y. *et al*. Overweight, resting heart rate, and prediabetes/diabetes: A population-based prospective cohort study among Inner Mongolians in China. *Sci. Rep*. **6**, 23939; doi: 10.1038/srep23939 (2016).

## Figures and Tables

**Table 1:** Baseline characteristics according to resting heart rate and overweight status in inner Mongolia, China.

	Heart rate <80 bpm Normal weight	Heart rate ≥80 bpm Normal weight	Heart rate <80 bpm Overweight	Heart rate ≥80 bpm Overweight	P value
n	935	461	210	123	
Age	44.1 ± 11.1	42.7 ± 11.2	44.1 ± 9.93	45.2 ± 8.93	0.041
Male, %	47.3	27.8	33.3	15.4	<0.001
Systolic blood pressure, mmHg	123.7 ± 20.9	125.1 ± 22.0	131.3 ± 22.4	136.0 ± 22.5	<0.001
Diastolic blood pressure, mmHg	81.6 ± 11.1	82.7 ± 12.7	86.7 ± 12.6	89.8 ± 13.2	<0.001
Total cholesterol, mmol/L	3.58 ± 0.99	3.64 ± 1.13	4.00 ± 1.17	3.84 ± 1.39	<0.001
Triglycerides, mmol/L	1.06 ± 0.90	1.17 ± 0.98	1.46 ± 1.13	1.69 ± 2.59	<0.001
HDL-cholesterol, mmol/L	1.20 ± 0.31	1.20 ± 0.34	1.11 ± 0.31	1.07 ± 0.32	<0.001
LDL- cholesterol, mmol/L	2.17 ± 0.90	2.21 ± 1.02	2.60 ± 1.09	2.43 ± 1.07	<0.001
Hyperlipidemia, %	27.9	33.0	47.1	47.2	<0.001
Smoker, %	48.9	39.3	32.9	23.6	<0.001
Drinker, %	35.1	25.4	29.0	16.3	<0.001
HOMA-IR	2.50 ± 1.29	2.90 ± 1.83	3.15 ± 1.71	3.39 ± 2.35	<0.001

HDL, high-density lipoprotein; LDL, low-density lipoprotein; HOMA-IR, homeostasis model assessment for insulin resistance.

**Table 2:** Age- and sex-adjusted and multivariable-adjusted odds ratios for prediabetes and diabetes incidence according to resting heart rate/overweight status.

	Cases	Age and sex adjusted		Multivariable adjusted*	
		Odds ratio	95% Confidence Interval	Odds ratio	95% Confidence Interval
Prediabetes	503				
Normal weight/Heart rate <80 bpm	239	1(reference)		1(reference)	
Normal weight/Heart rate ≥80 bpm	138	1.34	1.04–1.74	1.24	0.95–1.61
Overweight/Heart rate <80 bpm	76	2.12	1.51–2.97	1.83	1.29–2.61
Overweight/Heart rate ≥80 bpm	50	2.64	1.72–4.06	2.20	1.41–3.45
Diabetes	155				
Normal weight/Heart rate <80 bpm	56	1(reference)		1(reference)	
Normal weight/Heart rate ≥80 bpm	38	1.77	1.14–2.77	1.52	0.97–2.40
Overweight/Heart rate <80 bpm	37	4.91	3.05–7.92	3.64	2.21–6.01
Overweight/Heart rate ≥80 bpm	24	6.61	3.70–11.83	4.61	2.47–8.61

*Multivariable model adjusted for age, sex, smoking, drinking, systolic blood pressure, diastolic blood pressure, hyperlipidemia and HOMA-IR.

## References

[b1] Rosamond W. . Heart disease and stroke statistics–2008 update: a report from the American Heart Association Statistics Committee and Stroke Statistics Subcommittee. Circulation 117, e25–146 (2008). 18086926 10.1161/CIRCULATIONAHA.107.187998

[b2] Gu D. . Prevalence of diabetes and impaired fasting glucose in the Chinese adult population: International Collaborative Study of Cardiovascular Disease in Asia (InterASIA). Diabetologia 46, 1190–1198 (2003). 12879248 10.1007/s00125-003-1167-8

[b3] Yang W. . Prevalence of diabetes among men and women in China. N. Engl. J. Med. 362, 1090–1101 (2010). 20335585 10.1056/NEJMoa0908292

[b4] Report of the expert committee on the diagnosis and classification of diabetes mellitus. Diabetes care 26 (2003). 10.2337/diacare.26.2007.s5 12502614

[b5] Rathmann W. & Giani G. Global prevalence of diabetes: estimates for the year 2000 and projections for 2030. Diabetes Care 27, 2568–2569 (2004). 15451946 10.2337/diacare.27.10.2568

[b6] Kahn S. E., Hull R. L. & Utzschneider K. M. Mechanisms linking obesity to insulin resistance and type 2 diabetes. Nature 444, 840–846 (2006). 17167471 10.1038/nature05482

[b7] Shang X. . Educational level, obesity and incidence of diabetes among Chinese adult men and women aged 18–59 years old: an 11-year follow-up study. PLos One 8 (2013). 10.1371/journal.pone.0066479 PMC3688760 23840484

[b8] Abdullah A., Peeters A., de Courten M. & Stoelwinder J. The magnitude of association between overweight and obesity and the risk of diabetes: a meta-analysis of prospective cohort studies. Diabetes Res. Clin. Pract. 89, 309–319 (2010). 20493574 10.1016/j.diabres.2010.04.012

[b9] Yoon K. H. . Epidemic obesity and type 2 diabetes in Asia. Lancet 368, 1681–1688 (2006). 17098087 10.1016/S0140-6736(06)69703-1

[b10] Wu Y., Huxley R., Li M. & Ma J. The growing burden of overweight and obesity in contemporary China. CVD Prevention and Control 4, 19–26 (2009).

[b11] Shigetoh Y. . Higher heart rate may predispose to obesity and diabetes mellitus: 20-year prospective study in a general population. Am. J. Hypertens. 22, 151–155 (2009). 19151693 10.1038/ajh.2008.331

[b12] Grantham N. M. . Higher heart rate increases risk of diabetes among men: The Australian Diabetes Obesity and Lifestyle (AusDiab) Study. Diabet. Med. 30, 421–427 (2013). 23088496 10.1111/dme.12045

[b13] Zhang S., Tong W., Xu T., Wu B. & Zhang Y. Diabetes and impaired fasting glucose in Mongolian population, Inner Mongolia, China. Diabetes Res. Clin. Pract. 86, 124–129 (2009). 19712989 10.1016/j.diabres.2009.07.013

[b14] Perloff D. . Human blood pressure determination by sphygmomanometry. Circulation 88, 2460–2470 (1993). 8222141 10.1161/01.cir.88.5.2460

[b15] Sitzmann F. C. & Eschler P. [Enzymatic determination of blood glucose with a modified hexokinase method]. Med Klin 65, 1178–1183 (1970). 5517345

[b16] Allain C. C., Poon L. S., Chan C. S., Richmond W. & Fu P. C. Enzymatic determination of total serum cholesterol. Clin Chem 20, 470–475 (1974). 4818200

[b17] Friedewald W. T., Levy R. I. & Fredrickson D. S. Estimation of the concentration of low-density lipoprotein cholesterol in plasma, without use of the preparative ultracentrifuge. Clin. Chem. 18, 499–502 (1972). 4337382

[b18] Report of the Expert Committee on the Diagnosis and Classification of Diabetes Mellitus. Diabetes care 23 (2000). 12017675

[b19] Lehtovirta M. . Evidence that BMI and type 2 diabetes share only a minor fraction of genetic variance: a follow-up study of 23,585 monozygotic and dizygotic twins from the Finnish Twin Cohort Study. Diabetologia 53, 1314–1321 (2010). 20401462 10.1007/s00125-010-1746-4

[b20] Hartemink N., Boshuizen H. C., Nagelkerke N. J., Jacobs M. A. & van Houwelingen H. C. Combining risk estimates from observational studies with different exposure cutpoints: a meta-analysis on body mass index and diabetes type 2. Am. J. Epidemiol. 163, 1042–1052 (2006). 16611666 10.1093/aje/kwj141

[b21] Stevens J., Truesdale K. P., Katz E. G. & Cai J. Impact of body mass index on incident hypertension and diabetes in Chinese Asians, American Whites, and American Blacks: the People's Republic of China Study and the Atherosclerosis Risk in Communities Study. Am. J. Epidemiol. 167, 1365–1374 (2008). 18375949 10.1093/aje/kwn060 PMC2792196

[b22] Eckel R. H. . Obesity and type 2 diabetes: what can be unified and what needs to be individualized? J. Clin. Endocrinol. Metab. 96, 1654–1663 (2011). 21602457 10.1210/jc.2011-0585 PMC3206399

[b23] Eckel R. H. . Obesity and type 2 diabetes: what can be unified and what needs to be individualized? Diabetes Care 34, 1424–1430 (2011). 21602431 10.2337/dc11-0447 PMC3114323

[b24] Zhang X. . Resting heart rate and risk of type 2 diabetes in women. Int. J. Epidemiol. 39, 900–906, (2010). 20448009 10.1093/ije/dyq068 PMC2912487

[b25] Nagaya T., Yoshida H., Takahashi H. & Kawai M. Resting heart rate and blood pressure, independent of each other, proportionally raise the risk for type-2 diabetes mellitus. Int. J. Epidemiol. 39, 215–222 (2010). 19564246 10.1093/ije/dyp229

[b26] Carnethon M. R. . Resting heart rate in middle age and diabetes development in older age. Diabetes Care 31, 335–339 (2008). 17959868 10.2337/dc07-0874

[b27] Jamerson K. A., Julius S., Gudbrandsson T., Andersson O. & Brant D. O. Reflex sympathetic activation induces acute insulin resistance in the human forearm. Hypertension 21, 618–623 (1993). 8491496 10.1161/01.hyp.21.5.618

[b28] Brancati F. L. . Risk of end-stage renal disease in diabetes mellitus: a prospective cohort study of men screened for MRFIT. Multiple Risk Factor Intervention Trial. JAMA 278, 2069–2074 (1997). 9403420

[b29] Fuller J. H., Shipley M. J., Rose G., Jarrett R. J. & Keen H. Coronary-heart-disease risk and impaired glucose tolerance. The Whitehall study. Lancet 1, 1373–1376 (1980). 6104171 10.1016/s0140-6736(80)92651-3

[b30] Benjamin S. M., Valdez R., Geiss L. S., Rolka D. B. & Narayan K. M. Estimated number of adults with prediabetes in the US in 2000: opportunities for prevention. Diabetes care 26, 645–649 (2003). 12610015 10.2337/diacare.26.3.645

